# The moderating role of the late positive potential in the link between attachment anxiety and emotion regulation difficulties

**DOI:** 10.3389/fpsyg.2024.1360366

**Published:** 2024-11-13

**Authors:** Miguel Ramos-Henderson, Mónica Guzmán-González, Joaquín Bahamondes, Marcos Domic-Siede

**Affiliations:** ^1^Escuela de Psicología, Universidad Católica del Norte, Antofagasta, Chile; ^2^Centro de Investigación e Innovación en Gerontología Aplicada CIGAP, Facultad de Salud, Universidad Santo Tomás, Antofagasta, Chile

**Keywords:** adult attachment, emotion regulation, late positive potential, event-related potentials, cognitive reappraisal, expressive suppression

## Abstract

**Introduction:**

Understanding how adults experience and regulate their emotions is strongly linked to attachment orientations. Numerous studies indicate emotional regulation difficulties in both attachment avoidance and anxiety. Additionally, emotional Event-Related Potentials (ERPs), such as the Late Positive Potential (LPP), reveal the process of emotional information at the cerebral level, and thus, LPP is commonly used in studies examining emotion regulation processes. For instance, when individuals are asked to use cognitive strategies to increase, maintain, or decrease their emotional responses to stimuli, changes in LPP amplitude can reflect the effectiveness of these regulation strategies. However, little is known about the potential moderating effect of the LPP during the implementation of emotional regulation strategies in the relationship between attachment dimensions and emotional dysregulation. To address this oversight, the purpose of the present study was to examine the association between both dimensions of attachment, anxiety and avoidance, and emotional dysregulation, as well as the moderating role of the LPP during the induced implementation of cognitive reappraisal.

**Methods:**

Brain activity was recorded using EEG from *n* = 63 adults while they performed a task in which they were instructed to either reappraise or suppress emotions elicited by unpleasant images. To assess the associations between LPP, emotional dysregulation, and attachment orientations, the Difficulties in Emotion Regulation Scale Spanish version (DERS-E) and the Experiences in Close Relationships questionnaire (ECR-12) were used.

**Results:**

Interestingly, we found that greater LPP amplitudes during reappraisal implementation intensified the association between attachment anxiety and emotional regulation difficulties. Conversely, this relationship was non-significant under lower levels of LPP amplitude—Providing supporting evidence for the moderating role of LPP.

**Discussion:**

Our results highlight how attachment anxiety can influence the ability to regulate emotions. This study provides new insights into how variations in LPP contribute to the effectiveness of emotion regulation strategies.

## 1 Introduction

Attachment theory posits that humans are inherently motivated to form emotional bonds with others in pursuit of safety, comfort, and protection (Bowlby, 1989). Indeed, the attachment system is innate and functions to maintain closeness with significant others during times of stress or threat, thereby aiding in survival (Bowlby, 1982). Despite its innate nature, the quality of interactions with attachment figures in childhood is crucial in shaping internal working models (Bowlby, 1978), which encompass initial representations of oneself and others, influencing an individual’s affect, cognitions, and behaviors. These Internal Working Models (IWM) remain active throughout life and impact the quality of relationships with others (Mikulincer and Shaver, 2016).

The developmental trajectory and systematic pattern of relational expectations and behaviors, which arise from specific interactions with significant others, influence individuals’ levels of attachment security (Fraley and Shaver, 2000). In this context, research has identified various attachment styles, namely secure, anxious, and avoidant (Ainsworth et al., 1978). Additionally, another research strand (Brennan et al., 1998) suggests that both security and insecurity in adult attachment can be conceptualized along dimensions of attachment anxiety and avoidance. Within this framework, high levels of attachment anxiety and/or avoidance are associated with greater attachment insecurity. Conversely, attachment security is characterized by lower expressions in both of these dimensions (Brennan et al., 1998; Fraley et al., 2015).

The attachment anxiety orientation is characterized by a pronounced need for closeness, concerns about relationships, and fear of rejection (Brennan et al., 1998). Individuals with higher levels of attachment anxiety often feel undeserving of affection, care, or protection. This can lead to persistent doubts about their status in a relationship, stemming from a continual fear of abandonment (Mikulincer and Shaver, 2016). Such individuals tend to hyperactivate the attachment system, exaggerating their needs for proximity and support (Mikulincer et al., 2006).

Conversely, the attachment avoidance orientation manifests as insecurity stemming from the belief that others are unavailable in times of need. This belief may lead to compulsive self-reliance, a preference for emotional distance, and difficulties in trusting others (Mikulincer et al., 2006; Mikulincer and Shaver, 2016). The disruption in the sense of attachment security and the employment of secondary strategies such as attachment anxiety and/or avoidance (Main, 1990) are recognized as risk factors for psychopathology and emotional regulation issues (Cheche Hoover and Jackson, 2021; Trucharte et al., 2022).

Numerous studies have established links between individuals’ attachment orientations and emotional regulation strategies (Brandão et al., 2023; Domic-Siede et al., 2023a; Mikulincer and Shaver, 2019). Emotional regulation is understood as the processes through which we influence our emotions, determining when we experience them and how we experience and express these emotions (Gross and Ford, 2023). According to Gross and Ford (2023), each emotion is triggered by a situation that is psychologically significant to the individual. This implies a shift in attentional focus towards the emotional trigger. Subsequently, this leads to a cognitive-emotional appraisal of the situation, which then results in a response manifested at physiological, cognitive, and behavioral levels.

For individuals with an anxiety attachment orientation, increased rumination has been observed (Brandão et al., 2023), as well as a tendency to pay more attention to stressful stimuli (Silva et al., 2012). Conversely, individuals with a higher level of attachment avoidance orientation have reported greater emotional distancing in response to stressful events or stimuli (Holmberg et al., 2011; Shallcross et al., 2014).

Applying criteria of frequent usage in daily life, theoretical validity, operationalization, and manipulability in experimental conditions, Gross and colleagues (Gross, 2015; Gross and Ford, 2023) focus on two emotional regulation strategies: cognitive reappraisal and expressive suppression. Cognitive reappraisal is an antecedent-focused strategy aimed at altering the emotional meaning and impact of an emotion-eliciting situation (Gross and John, 2003; Guzmán-González et al., 2020b). In contrast, suppression, a response modulation strategy, is defined as the inhibition of emotional expression (Gross, 1998). This occurs after the emotion has been generated and thus does not influence the emotion itself but rather its outcomes.

In general, the use of cognitive reappraisal is positively associated with well-being (Gross and John, 2003), negatively with psychopathology (Aldao et al., 2010), and serves as a moderator between stressors and negative outcomes. As such, it operates as a proximal factor for resilience (Riepenhausen et al., 2022). On the contrary, the use of expressive suppression is linked with increased depressive symptoms and lower satisfaction in interpersonal relationships (Gross and John, 2003; Srivastava et al., 2009) and even with higher mortality risk in 12-year follow-up studies (Chapman et al., 2013).

Within the context of attachment, individuals reporting greater attachment security (low anxiety and avoidance) experience fewer emotional control difficulties, contrasting with the lack of control, daily interference, and emotional rejection reported by those with high levels of attachment anxiety (Guzmán-González et al., 2016).

Regarding emotion regulation, individuals with higher levels of attachment anxiety exhibit difficulties in accessing effective strategies and controlling impulses, as well as in identifying and accepting their emotions (Henschel et al., 2020). In the case of a greater orientation towards attachment avoidance, it has been suggested that such individuals are more likely to deactivate their attachment system (Mikulincer and Shaver, 2016). In this vein, some authors (Vrticka and Vuilleumier, 2012) have reported a predominant use of emotion suppression in these individuals and less efficiency in reappraising unpleasant emotions. Additionally, high levels of attachment avoidance have been reported to act as a moderator in the relationship between the use of suppression and depressive symptomatology (Brandão et al., 2022). The attachment anxiety and avoidance dimensions are closely related to the effectiveness of implementing emotion regulation strategies and their respective impacts (Guzmán-González et al., 2020a).

Emotions and their regulation are psychological, social, and biological processes underpinned by specific changes in the brain and nervous system (Matsumoto and Hwang, 2012; Šimić et al., 2021). In this context, the amygdala, insula, and anterior cingulate cortex play crucial roles in the detection of emotions (Phan et al., 2002). These structures form a processing network with other regions that are more advanced in phylogenetic development, such as the dorsolateral, ventrolateral, and orbitofrontal prefrontal cortices (Berboth and Morawetz, 2021; Lévesque et al., 2003; Ochsner et al., 2004). This network facilitates more complex aspects, such as appraisal and cognitive reappraisal, as well as the top-down and bottom-up regulation of emotions (Bastiaansen et al., 2018).

In relation to attachment, some researchers (Zhang et al., 2018) have identified morphometric differences in the brains of individuals with anxious and avoidant attachment orientations. Specifically, they observed a smaller size in the left medial temporal gyrus and right parahippocampal gyrus in individuals with higher attachment avoidance and a smaller volume in the right anterior cingulate cortex in those with higher attachment anxiety. A systematic review (Perlini et al., 2019) reported correlations between different attachment orientations and the volume of the hippocampus, amygdala, and anterior temporal pole, as well as activation patterns in fronto-striatal-limbic circuits during the processing of attachment-related social stimuli. More specifically, these individuals have shown more intense emotional responses, with greater activity in brain regions typically associated with distress, such as the insula and anterior cingulate cortex (DeWall et al., 2012; Stevens et al., 2011). In the case of individuals with higher attachment avoidance, increased activity in the amygdala and right dorsolateral prefrontal cortex has been observed during cognitive reappraisal, accompanied by difficulties in reducing arousal and discomfort in unpleasant social scenarios (Vrtička et al., 2014; Winterheld, 2016).

Emotion regulation and its relationship with attachment have been studied in experimental contexts, utilizing brain morphology analysis, functional Magnetic Resonance Imaging (fMRI), as well as less invasive and cost-effective but high temporal resolution techniques like Electroencephalography (EEG) (Eilert and Buchheim, 2023). In this domain, Event-Related Potentials (ERPs) represent significant voltage changes at specific times in relation to an event or stimulus (Helfrich and Knight, 2019). Late ERPs, particularly those occurring after 150 ms following the presentation of an emotional stimulus, are critical in emotional regulation research as they provide information about how emotion is regulated or modulated over time (MacNamara et al., 2022).

Specifically, the Late Positive Potential (LPP), which emerges around 300 ms after the onset of an emotional stimulus and in response to emotionally intense stimuli of both unpleasant and pleasant valence, shows increased amplitude compared to neutral stimuli. This highlights its role in the extended cognitive processing of emotional stimuli (Cuthbert et al., 2000). Due to its duration, the LPP is also considered a biological marker of emotion regulation (Dennis and Hajcak, 2009; Hajcak et al., 2010; Kennedy and Montreuil, 2021). The amplitude of the LPP tends to decrease during the process of cognitive reappraisal (Harrison and Chassy, 2019). Similarly, during emotional suppression, a reduction in LPP amplitude has also been observed in response to unpleasant stimuli (Liu et al., 2012; Moser et al., 2006). Notably, emerging evidence suggests that the amplitude of the LPP, while traditionally associated with the intensity of emotional engagement, may exhibit a complex modulation based on the nature of the emotion regulation strategy employed and the characteristics of the eliciting stimuli. For instance, Foti and Hajcak (2008) demonstrated that the neural response to emotionally arousing pictures can be modulated by descriptions provided before the stimuli, indicating that the cognitive framework set by reappraisal strategies can influence the subsequent LPP amplitude. Similarly, Gan et al. (2015) explored the electrocortical modulation effects of different emotion regulation strategies, finding that the specific strategy employed significantly affects the LPP response, which underscores the adaptability of the brain’s emotional processing systems to the regulatory goals. Further expanding on this, Del Popolo Cristaldi et al. (2022) highlighted the differential modulation of neural activity across various stages of affective prediction, suggesting that the anticipatory phase of emotion regulation plays a crucial role in shaping the LPP response. This points towards an intricate relationship between the type of emotion regulation strategy, the temporal dynamics of its application, and the resulting neural signatures. Myruski et al. (2019) provided additional insights by proposing the LPP as a neurocognitive index of emotion regulatory flexibility, demonstrating that the adaptability in employing various emotion regulation strategies is mirrored in the variability of LPP amplitudes across different contexts.

These findings collectively suggest a more nuanced perspective on the role of the LPP in emotion regulation. It appears that the LPP amplitude is not merely reflective of the emotional intensity of a stimulus but is also significantly shaped by the emotion regulation strategy employed and the specific characteristics of the stimulus, including its arousal level and predictability. This evidence shows the importance of considering the context-dependent nature of LPP modulation when evaluating its role as a biological marker of emotion regulation.

It is well-established that individuals with high levels of attachment avoidance and/or anxiety exhibit difficulties in regulating their emotions (Cheche Hoover and Jackson, 2021; Rogier et al., 2023). Additionally, late ERPs such as the LPP are recognized as indices of emotional reactivity, which can be modulated via emotional regulation at the brain level (MacNamara et al., 2022). However, the potential moderating effect of the LPP during the implementation of the emotion regulation reappraisal strategy in the relationship between attachment dimensions and emotional dysregulation remains less understood.

In this context, the aim of the current study was to analyze the relationship between the attachment orientations of anxiety and avoidance with emotion regulation difficulties and to explore the potential moderating effect of the LPP during the induced implementation of cognitive reappraisal strategy.

## 2 Materials and methods

Our study utilizes a design approach that combines experimental, correlational, and moderation methodologies to investigate the moderating role of neural emotional processing in the link between attachment orientations and emotion regulation.

For this study, the dimensions of attachment (anxiety and avoidance) were assessed using the Experiences in Close Relationships-12 questionnaire (Brennan et al., 1998; Guzmán-González et al., 2020a). Emotional intensity was measured through the LPP component, and scores were obtained from the Self-Assessment Manikin (SAM) scale (Bradley and Lang, 1994) during an emotion regulation task. To assess the level of emotion dysregulation in participants, the Chilean version of the Difficulties in Emotion Regulation Scale (DERS-E) (Gratz and Roemer, 2004; Guzmán-González et al., 2014; Hallion et al., 2018) was used. The chosen instruments were selected for their specific relevance to the constructs being studied—attachment and emotion regulation—and their validated use within the Chilean population. The combination of these instruments allows for a comprehensive and culturally appropriate assessment of the key variables.

### 2.1 Participants

Individuals over the age of 18 who were right-handed (to control for potential variations in brain structure and function associated with handedness) and had normal or corrected vision were included in the study. Exclusion criteria involved individuals with neuropsychiatric conditions where reality testing was compromised. We employed a non-probabilistic purposive sampling method to specifically target individuals who met our inclusion criteria. The sample was recruited through a call for voluntary participation using the official digital platforms of the School of Psychology and the Faculty of Humanities at the Universidad Católica del Norte in Antofagasta, Chile. All participants voluntarily signed an informed consent form before participating in the experiment. There was no financial compensation for participation. A total of 63 individuals participated, with ages ranging from 18 to 58 years (mean = 28.22; SD = 9.87). The majority were female (*n* = 32, 50.8%), with incomplete higher education (*n* = 36, 57.1%), single (*n* = 51, 81%), and unemployed (*n* = 32, 50.8%). Clinically, most participants were not undergoing psychiatric treatment (*n* = 55, 87.3%) or psychotherapy (*n* = 52, 82.5%) and did not have chronic and/or mental health illnesses (see Table 1 and Supplementary Table S1). The sample size was carefully considered using the G*Power 3.1.9.2 software^1^. This process was based on the parameters of the ANOVA test (Repeated measures, within factors), incorporating an effect size of 0.25, an alpha value of 0.05, and aiming for a power of 0.80 (Faul et al., 2007).

**Table 1:** Sociodemographic characteristics of participants.

	Total participants (*n*) = 63	
Variables	*n*	%
Sex		
Female	32	50.8
Male	31	49.2
Education		
Complete High School	5	7.9
Complete Technical Education	7	11.1
Incomplete Higher Education	36	57.1
Complete University Education	8	12.7
Postgraduate	7	11.1
Marital status		
Single	51	81.0
Married	11	17.5
Other	1	1.6
Occupation		
Unemployed	32	50.8
Homemaker	1	1.6
Employed	30	47.6
Chronic Illness		
Yes	20	31.7
Mental health disorder		
Yes	15	23.8
Psychotherapy		
Yes	11	17.5
Psychiatric treatment		
Yes	8	12.7

The research received approval from the Scientific Ethics Committee (CEC) of the Universidad Católica del Norte under resolutions No. 099/2021 and No. 037/2023.

### 2.2 Instruments

#### 2.2.1 Experiences in close relationships questionnaire (ECR-12)

The ECR is a questionnaire developed by Brennan et al. (1998) to measure adult attachment, considering two dimensions: attachment anxiety and avoidance. For this research, the 12-item short version adapted for the Chilean population was used (Guzmán-González et al., 2020b). This version features two subscales: attachment avoidance (e.g., “*I feel uncomfortable opening up to my partner*”) and attachment anxiety (e.g., “*If I cannot get my partner to show interest in me, I get angry or upset*”), each containing six items. Responses were recorded using a 7-point Likert scale, ranging from (1), indicating strong disagreement, to (7), representing strong agreement. The results indicate high or low levels of attachment anxiety and/or avoidance based on the average scores obtained on each item. This questionnaire has shown good psychometric properties in terms of reliability and construct validity, accurately reflecting the two theoretical dimensions (anxiety and avoidance) proposed in the original version by Brennan et al. (1998) and the psychometric and reliability properties of the Chilean version of the 36-item ECR (Spencer et al., 2013). In the present study, the reliability of the subscales was assessed using Cronbach’s alpha. The Attachment Avoidance subscale yielded a Cronbach’s alpha of 0.78, and the Attachment Anxiety subscale showed a Cronbach’s alpha of 0.79, indicating good internal consistency for both dimensions.

#### 2.2.2 Difficulties in emotion regulation scale Spanish version (DERS-E)

The Difficulties in Emotion Regulation Scale (DERS), developed by Gratz and Roemer (2004), is designed to assess problems in emotion regulation through self-report. For this study, the Spanish and Chilean-validated version, DERS-E (Guzmán-González et al., 2014), was used. It consists of 25 items in a 5-point Likert format (1 = Almost never, 5 = Almost always), where higher scores indicate greater difficulties in emotion regulation. The Chilean version contains five subscales: Emotional Rejection (Nonacceptance of Emotional Responses), Lack of Emotional Control (Impulse Control Difficulties and Limited Access to Emotion Regulation Strategies), Emotional Interference (Difficulties Engaging in Goal-Directed Behavior), Emotional Inattention (Lack of Emotional Awareness), and Emotional Confusion (Lack of Emotional Clarity). This scale has shown good psychometric properties in terms of its construct validity and reliability indices (Hallion et al., 2018). For examples of items for each dimension and more explanation about what each dimension measures, see Supplementary Table S2. The overall reliability of the scale in this study was high, with a Cronbach’s alpha of 0.91 for the DERS-Total score.

#### 2.2.3 State–trait anxiety inventory (STAI)

The State–Trait Anxiety Inventory (STAI), developed by Spielberger et al. (1983), is a widely used self-report instrument designed to measure both state and trait anxiety. The STAI comprises two separate subscales: the State Anxiety Scale (S-Anxiety) and the Trait Anxiety Scale (T-Anxiety). Each subscale contains 20 items, resulting in a total of 40 items.

- State Anxiety (S-Anxiety): This subscale assesses how the respondent feels “right now, at this moment,” capturing the transient and situational aspects of anxiety. Items are rated on a 4-point Likert scale ranging from (1) “Not at all” to (4) “Very much so.” Example items include “I feel calm,” “I am tense,” and “I am worried.”
- Trait Anxiety (T-Anxiety): This subscale measures the respondent’s general and long-standing anxiety disposition. Items are also rated on a 4-point Likert scale, but the responses range from (1) “Almost never” to (4) “Almost always.” Example items include “I feel nervous and restless,” “I worry too much over something that really does not matter,” and “I am content.”

Responses on both subscales are summed to yield separate scores for state and trait anxiety, with higher scores indicating greater anxiety levels. The STAI has demonstrated excellent psychometric properties, with strong internal consistency and test–retest reliability for both subscales. It also shows good construct validity, accurately distinguishing between state and trait anxiety.

For this research, the Spanish adaptation of the STAI was utilized (Spielberger et al., 2002). This version maintains the original structure and has shown comparable psychometric properties, making it a reliable tool for assessing anxiety in diverse cultural contexts. This instrument was used in our study to control for confounding variables related to anxiety in the analysis.

### 2.3 Experimental paradigm

To evaluate emotion regulation, we implemented an experimental task detailed in Domic-Siede et al. (2023a), adapted from tasks used in previous studies (Ochsner et al., 2004; Schlumpf et al., 2019; Vrticka and Vuilleumier, 2012). The task programming was executed using Presentation® software by Neurobehavioral Systems (Version 18.0^2^, Neurobehavioral Systems, Inc., Albany, CA). A selection of 60 images from the International Affective Picture System (IAPS) (Lang et al., 2005) was chosen. This selection included 45 images with unpleasant emotional content and 15 neutral images. Participants were exposed to these images under three distinct experimental conditions: “Natural” (15 unpleasant and 15 neutral images), “Reappraise” (15 unpleasant images), and “Suppress” (15 unpleasant images) (Domic-Siede et al., 2023a).

The images for the “Natural,” “Reappraise,” and “Suppress” conditions were chosen based on prior research utilizing this emotion regulation experimental paradigm (Domic-Siede et al., 2023a; Domic-Siede et al., 2024a; Domic-Siede et al., 2024b; Moser et al., 2009; Schlumpf et al., 2019), as well as the valence and arousal ratings reported in the IAPS study (Lang et al., 2005). For a detailed list of the specific IAPS images selected in the study, please refer to Supplementary Table S3. Supplementary Table S4 provides the descriptive statistics of the selected images based on their valence and arousal ratings. Additionally, we conducted a Tukey test (Supplementary Table S5) to confirm that the IAPS pictures chosen for each condition (“Natural-neg,” “Suppress,” and “Reappraise”) were equivalent in terms of valence and arousal, and distinct from the “Natural-neu” condition. This analysis ensured that the selected images were suitable for use in our emotion regulation paradigm.

The stimuli were displayed on a 23.6” ASUS VG248QE LCD monitor positioned 82 cm from the subject. Participants were required to evaluate the intensity (arousal) of their emotions while viewing emotional (unpleasant) or neutral images. Before beginning, an experimenter explained the task using visual aids, ensuring participants understood the instructions and the purpose of each condition. Then, participants underwent a training session to familiarize themselves with the experimental setup and the objectives of each trial condition. This session consisted of 3 blocks, with 3 trials for each condition.

For the “Natural” instruction, participants were asked to actively observe the image and pay attention to their evoked emotion, trying to engage with what they observed. In the “Reappraise” condition, participants were required to look at the image and, through cognitive reappraisal strategies, attempt to diminish the emotional impact by attributing a different meaning to it. For example, they could imagine that the situation was fictional and that the people in the image were actors or actresses or envision a positive outcome for the situation depicted in the image. Participants were previously trained in cognitive reappraisal strategies using examples with images presented in a slideshow. This training included both self-focused and situation-focused reappraisal techniques (Dunne et al., 2018; Ochsner et al., 2004). To ensure participants understood the reappraisal strategies, they were asked to describe how they implemented these strategies during the training session. For the “Suppress” instruction, participants were asked to observe the image and regulate any emotional response by avoiding any external expression of the emotion-elicited situation. Training for expressive suppression included a demonstration of a neutral facial expression (Dunne et al., 2018; Ochsner et al., 2004). Participants’ understanding of expressive suppression was also verified by asking them to explain how they implemented suppression strategies during the training.

After viewing each image according to the assigned instruction (“Natural,” “Reappraise,” and “Suppress”), participants assessed the emotion experienced using a 1 to 7 Likert scale, employing the Self-Assessment Manikin (Bradley and Lang, 1994) to measure their arousal. The arousal ratings ranged from 1 (low intensity) to 7 (high intensity), as well.

**Figure 1:**
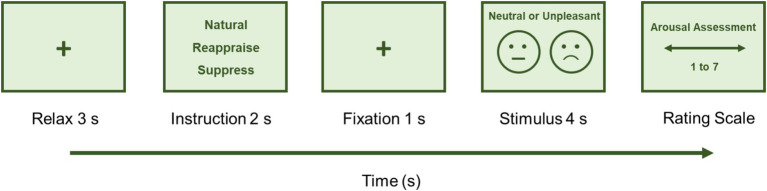
Emotion regulation task. The figure depicts the sequence of the emotion regulation task designed to measure the participants’ neural and psychological responses to stimuli. The task is time-sequenced as follows: (1) Relax: Participants begin with a relaxation period, signified by a fixation cross displayed for 3 s. This allows the participants to focus and prepare for the task ahead. (2) Instruction: Next, they receive instructions for 2 s, indicating the regulation strategy to be employed: ‘Natural,’ ‘Reappraise,’ or ‘Suppress.’ This informs participants how to approach the upcoming stimulus. (3) Fixation: Another fixation cross is then shown for 1 s, serving as a consistent visual cue to maintain the participant’s focus. (4) Stimulus: A stimulus, which can be either neutral or unpleasant (Natural Neutral) or unpleasant only (Natural Negative, Reappraise, and Suppress conditions), is presented for 4 s. The emotional content of the stimulus is intended to evoke a response that participants must regulate (or not) according to the previous instruction. (5) Rating Scale: Following the stimulus presentation, participants assess their arousal level using a SAM rating scale from 1 to 7, where 1 indicates the lowest arousal and 7 indicates the highest.

The experimental session was structured in a random sequence of 12 blocks. Each block consisted of five images corresponding to one of the three conditions: “Natural,” “Reappraise,” and “Suppress.” Specifically, the “Natural” condition included a total of 30 trials, with 15 unpleasant and 15 neutral images. The “Natural” condition was divided into two categories: “Natural-neu” for neutral images and “Natural-neg” for unpleasant images. The “Reappraise” and “Suppress” conditions consisted of 15 trials each, exclusively with unpleasant images. Each block followed this sequence, as shown in Figure 1: (1) A gray background with a fixation cross “+” for 3 s to orient the participant’s attention; (2) The cue task instruction, specifying the condition of the block (“Natural,” “Reappraise,” or “Suppress”) displayed for 2 s; (3) A fixation cross reappeared for 1 s in the center of the screen; (4) The image (unpleasant or neutral) was displayed for 4 s; (5) Finally, the SAM scale screen was presented, allowing participants to manually rate the arousal level of the experienced emotion using a computer mouse. The images within each block were also randomized to further minimize any potential habituation effects. This procedure was repeated in each block until all were completed. At the end of each block, an interlude was displayed on the screen, offering a brief pause before moving to the next block. The pause ended when subjects pressed a button. The entire experiment lasted approximately 30 min.

### 2.4 EEG data acquisition

Brain activity was recorded using an ANT-Neuro electroencephalography system^3^, comprising 30 channels positioned on the scalp and two on the mastoids, following the international 10/20 system. Each electrode was placed according to anatomical norms (Keil et al., 2014). Data were sampled online at a rate of 1,024 Hz and referenced to the average of the mastoids. Impedance for all channels was maintained at less than 20 kΩ throughout the recording session.

### 2.5 Data analysis

Our analytical approach employs a combination of experimental, correlational, and moderation designs to comprehensively investigate the interplay between emotion regulation strategies, attachment orientations, and neural responses.

#### 2.5.1 Behavioral data analysis

Descriptive statistics and hypothesis tests estimations were performed using the Jamovi statistical software 2.3 version (Jamovi Project, 2023). The Shapiro–Wilk normality test was conducted to decide whether to use parametric or non-parametric statistical hypothesis tests (Mishra et al., 2019). Friedman tests were conducted to analyze differences between emotional regulation conditions, and *post hoc* tests with multiple comparison corrections (Durbin-Conover) were employed and Cohen’s *d* effect sizes were calculated to quantify the magnitude of the differences observed between conditions.

The primary dependent variable in our behavioral analysis was the arousal rating provided by participants after each trial, measured on a Likert scale from 1 (low arousal) to 7 (high arousal). The independent variable included Emotion Regulation Conditions: A within-subjects factor with four levels: “Natural Negative,” “Natural Neutral,” “Reappraise,” and “Suppress.” The experimental design was a within-subjects design, with participants exposed to each level of the independent variables.

Additionally, correlation analyses were carried out using the Spearman coefficient to determine the degree of association between the values of arousal ratings and LPP amplitudes obtained during the emotion regulation task, the two attachment orientations (ECR-12) and emotional regulation difficulties (DERS-E). These latter analyses were conducted using the JASP statistical software (JASP Team, 2023). To address the issue of multiple comparisons, a False Discovery Rate correction was applied (Benjamini and Hochberg, 1995).

Furthermore, we conducted Spearman correlation analyses between the ECR-12 Avoidance and Anxiety Attachment scales and both the STAI-T and STAI-S scores. These analyses were conducted to explore potential correlations that could confound the relationships we were examining between attachment orientations and the variables of interest. The results of these analyses revealed no significant correlations between the ECR-12 Avoidance and Anxiety scales and the STAI-T and STAI-S scores (Supplementary Table S6). This finding suggests that the attachment dynamics we observed in our study are not merely reflections of underlying traits or state anxiety levels.

#### 2.5.2 EEG signal preprocessing

The procedure began by applying dual filtering techniques: a high-pass Finite Impulse Response (FIR) filter set at 0.5 Hz to remove slow drifts, and a low-pass filter with a cutoff at 20 Hz to eliminate high-frequency noise. Following, the signal was downsampled to a rate of 256 Hz and divided into 15 separate trials for each of the conditions: “Natural-neu,” “Natural-neg,” “Suppress,” and “Reappraise.” In each trial, a period of one second preceding the onset of either emotional or neutral image presentations and a subsequent duration of 4 s were included for analysis.

A visual inspection was initially performed to identify and discard trials with evident artifacts. We conducted a Friedman test and *post hoc* Dunn test to assess whether there were differences in the final number of trials between the conditions (“Natural Negative,” “Natural Neutral,” “Reappraise,” “Suppress”) (Supplementary Tables S7–S9). Next, the Independent Component Analysis (ICA) Logistic Infomax algorithm (Bell and Sejnowski, 1994) was applied to identify and remove EEG artifact components, such as blinks, eye movements, jaw muscle noise, line noise, and heartbeats. This stage was facilitated using the ICLabel plugin (Pion-Tonachini et al., 2019). The artifact removal process was semi-automated and validated by visual inspection, where each component, including those not labeled by ICLabel, was examined. This involved considering the spectrum of the component, the scalp distribution, and the component behavior across time and trials. Components identified as artifacts were discarded. As a final step, channels exhibiting noise, identified both visually and through their frequencies, were subjected to spherical interpolation (Delorme and Makeig, 2004; Perrin et al., 1989). The preprocessing pipeline followed the protocols established by Domic-Siede et al. (2021) and Domic-Siede et al. (2023b).

#### 2.5.3 Analysis of event-related potential: LPP

The segmented signal was corrected using a pre-stimulus interval of 1,000 ms on an intra-subject level. The signal was then averaged for each of the four conditions for each channel and subject. Analyses of the LPP component were performed on the mean ERP amplitudes within electrode groups of a region of interest (ROI): POz, P3, Pz, and P4. The measurement time windows and electrodes selected for the ROI were based on previous studies and on the visual inspection of the signal in this study (250 ms to 800 ms) (Cuthbert et al., 2000; Hajcak et al., 2010; MacNamara et al., 2009; MacNamara et al., 2022). Additionally, mean amplitude values for each temporal window corresponding to each condition were extracted.

The dependent variable in our ERP analysis was the amplitude pf the LPP. The independent variables were Emotion Regulation Conditions in a within-subjects factor with four levels: “Natural Negative,” “Natural Neutral,” “Reappraise,” and “Suppress.” This, the experimental design was a within-subjects design.

ERPs from the ROI were compared between conditions using the Friedman test and *post hoc* tests with corrections for multiple comparisons using the Durbin-Conover test. Spearman correlation analyses were also conducted between the LPP amplitude, the scores from the emotion regulation task (arousal levels for each condition), the dimensions of attachment avoidance and anxiety, as measured by the ECR-12 scale, and the Difficulties in Emotion Regulation Scale (DERS).

#### 2.5.4 Moderation analysis

**Figure 2:**
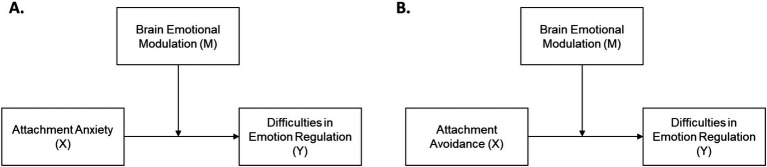
Moderation model. The moderation model highlights how the brain’s modulation of emotional responses (M), as reflected in the Late Positive Potential (LPP), potentially influences the relationship between attachment anxiety (A) or attachment avoidance (B) (the predictor variables X), and difficulties in emotion regulation in daily life (the dependent variable Y).

Finally, after checking de fulfillment of assumptions of linearity, homoscedasticity, and multicollinearity, moderation analyses were conducted using the dimensions of attachment (avoidance and anxiety) as focal predictors. The dimensions of the DERS-E were used as dependent variables, and the amplitude of LPP during the “Reappraise” condition, as recorded during the experimental task, was used as the moderating variable. As a control, we also tested the model using the “Suppress” condition (Supplementary Tables S10, S11). The Johnson-Neyman technique and plot (D’Alonzo, 2004; Johnson and Fay, 1950; Lin, 2020) were utilized to identify and visualize the conditional threshold or the moderated effect. Predictors were mean-centered to facilitate interpretation of parameter estimates. The moderation model and its variables are described in Figure 2.

## 3 Results

Firstly, we calculated the average scores of the ECR-12 and the dimensions of the DERS-E scale (Table 2). Similarly, we obtained the mean values of the LPP (microvolts, μV) and the levels of arousal reported during the neutral natural, negative natural, reappraise, and suppress conditions.

**Table 2:** Descriptive statistics of ECR-12, DERS-E, and late positive potential (LPP) in μV.

	*N* = 63			
	*M*	SD	SEM	CI 95%
ECR-12 avoidance (ECR-12-AVD)	2.36	1.06	0.13	2.09–2.63
ECR-12 anxiety (ECR-12-ANX)	3.51	1.25	0.16	3.19–2.82
DERS-E emotional rejection (DERS-ER)	17.56	8.27	1.04	15.47–19.64
DERS-E lack of emotional control (DERS-LEC)	13.33	6.14	0.77	11.78–14.88
DERS-E emotional interference (DERS-INT)	11.48	5.08	0.64	10.19–12.76
DERS-E emotional inattention (DERS-INATT)	13.06	4.41	0.55	11.95–14.18
DERS-E emotional confusion (DERS-EC)	7.46	2.75	0.34	6.76–8.15
DERS-E total (DERS-T)	62.89	19.01	2.39	58.10–67.68
Arousal natural neutral (Arousal-Nneu)	2.06	0.97	0.12	1.87–2.31
Arousal natural negative (Arousal-Nneg)	2.92	1.37	0.17	2.56–3.26
Arousal reappraise (Arousal-Reapp)	2.42	1.21	0.15	2.11–2.73
Arousal suppress (Arousal-Supp)	2.59	1.30	0.16	2.26–2.92
LPP ROI natural negative (LPP-Nneg)	2.13	2.31	0.29	1.54–2.71
LPP ROI natural neutral (LPP-Nneu)	1.26	1.87	0.23	0.79–1.73
LPP ROI reappraise (LPP-Reapp)	2.07	2.53	0.32	1.42–2.71
LPP ROI suppress (LPP-Supp)	2.30	2.45	0.31	1.68–2.92

M, mean; SD, standard deviation; SEM, standard error of mean; CI, confidence interval.

**Figure 3:**
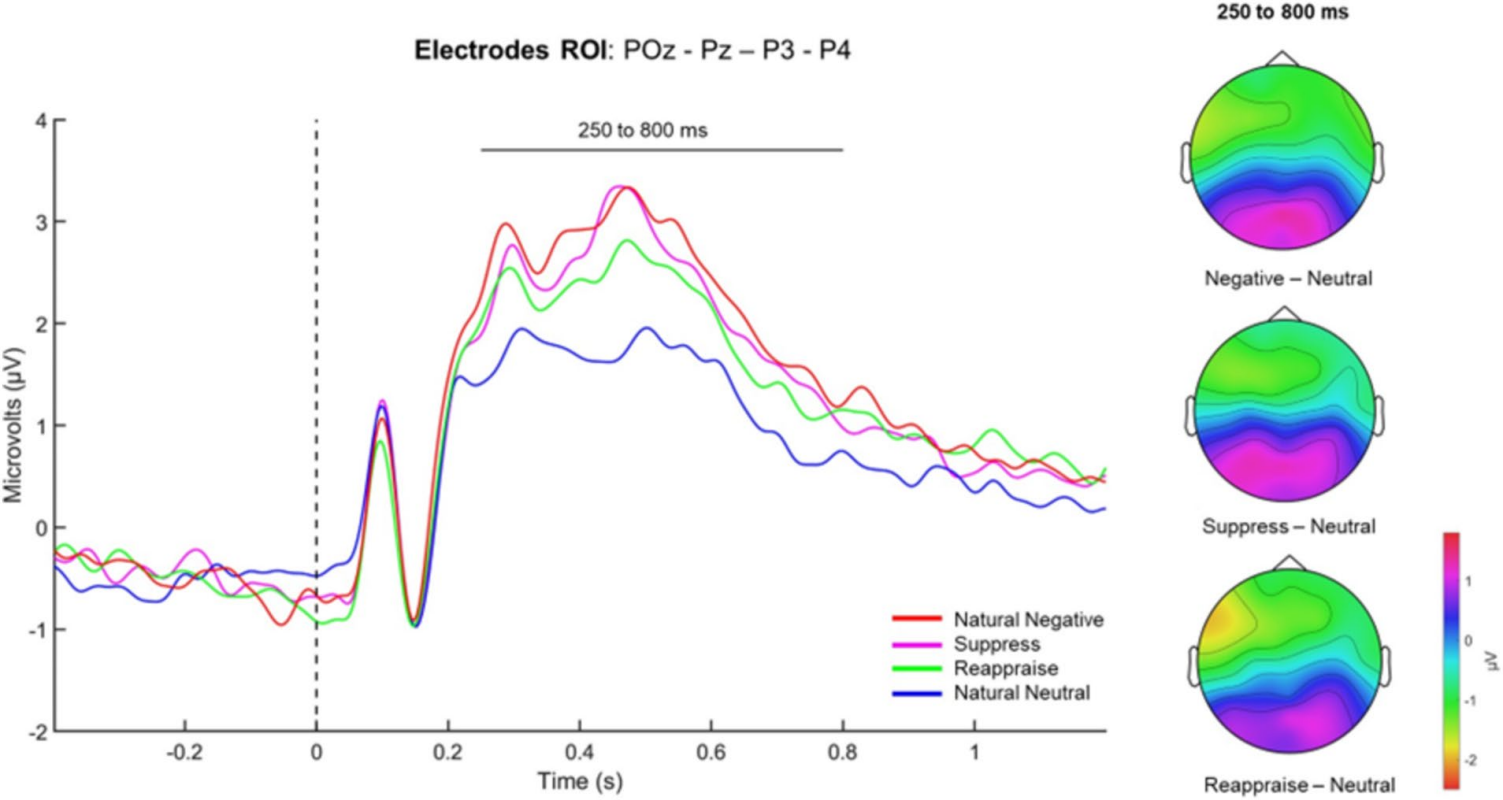
Late positive potential (LPP) during the emotion regulation task. The graph presents a time-course plot of the LPP measured at posterior electrode sites of interest (POz, Pz, P3, and P4) during the emotion regulation task. The LPP waveforms are plotted as microvolts (μV) against time in seconds (s), with a period of −400 to 1,500 milliseconds relative to the stimulus onset. The LPP is quantified during four distinct conditions of emotional regulation: Natural Negative, Suppress, Reappraise, and Natural Neutral, each depicted by a different colored line on the graph. The waveform associated with the Natural Negative condition (red line) peaks higher than the other conditions, indicating a more substantial emotional response to negative stimuli. The Reappraise condition (green line) shows a moderately elevated response, suggesting an intermediate level of emotional arousal when participants are instructed to cognitively reappraise the emotional content. The Suppress condition (purple line) demonstrates a response pattern that lies between the Natural Negative and Reappraise conditions, reflecting the emotional response during attempts to suppress the emotional expression. The Natural Neutral condition (blue line) exhibits the lowest response, consistent with the expected minimal emotional arousal elicited by neutral stimuli. The critical measurement window from 250 to 800 ms post-stimulus is highlighted, where the LPP is most prominent. This window is used to analyze the differences in brain activity associated with emotional processing across conditions. Topographical maps in the inset illustrate the scalp distribution of the LPP amplitude during the 250 to 800 ms window, across conditions. These maps show the concentration of activity at the posterior sites, with warmer colors indicating higher amplitude responses, corresponding to greater emotional engagement. Overall, a qualitative inspection of the graph and topographical maps collectively suggest that the processing of emotional stimuli, as modulated by task instructions, is reflected in the LPP plots across different emotional regulation conditions. The statistical analysis, as indicated by the Friedman test and Durbin-Conover pairwise comparisons, provides evidence for significant differences in LPP amplitudes between specific conditions (Natural Negative, Reappraise, and Suppress vs. Natural Neutral).

Figure 3 illustrates the average cerebral response of the LPP (μV) during the emotional regulation task across the four experimental conditions: Natural Negative (LPP ROI-Nneg), Natural Neutral (LPP ROI-Nneu), Reappraise (LPP ROI-Reapp), and Supp (LPP ROI-Supp). As observed, the LPP amplitude increases in the presence of negative stimuli (red line), which is reduced in the natural neutral condition (blue line). The Friedman test confirms that these differences were significant [χ2 = 46.5(3); *p* < 0.001].

The Durbin-Conover pairwise comparison revealed differences between the LPP ROI-Nneg and LPP ROI-Nneu conditions (*T* = 6.55; *p* < 0.001; *d* = 0.41), LPP ROI-Nneu and LPP ROI-Reapp (*T* = 5.48; *p* < 0.001; *d* = −0.36), and between LPP ROI-Nneu and LPP ROI-Supp (*T* = 6.75; *p* < 0.001; *d* = −0.48). No differences were found among the other pairs of comparisons.

While the statistical tests did not show significant differences between the LPP amplitudes in the reappraise and suppress conditions, visual inspection of the waveforms suggests slight variations. Specifically, a lower amplitude of LPP can be observed during reappraisal compared to the Suppress and Natural Negative conditions. These qualitative comments are based on the visual inspection of the plots and are not supported by statistical analysis.

Similarly, the topographical maps provide a visual representation of the distribution of LPP amplitude across the scalp within the 250–800 ms post-stimulus interval for each condition. These depictions reveal a focal accumulation of activity at posterior electrode locations, with more intense hues showing elevated amplitude responses indicative of heightened emotional involvement. Overall, these data may indicate that the modulation of emotional stimuli processing—guided by the task’s instructions—is manifested in the differential amplitudes of the LPP across the examined emotion regulation conditions.

Therefore, correlation and moderation analyses were conducted to determine if there is an association and potential effects of the LPP in these putative relationships.

**Figure 4:**
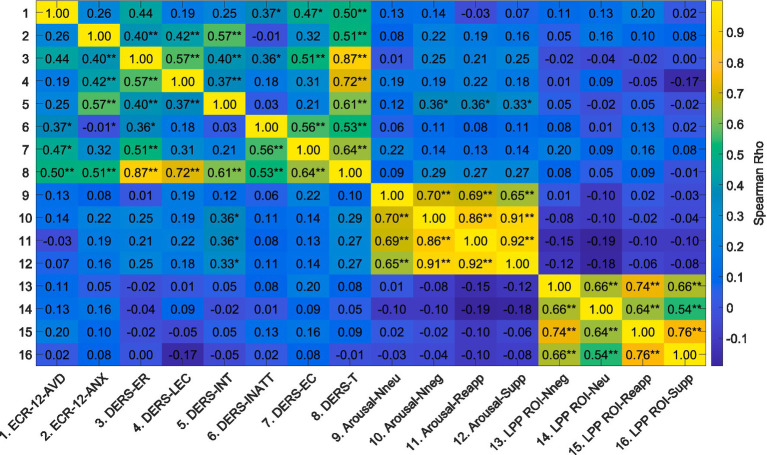
Spearman correlations between attachment dimensions (ECR-12), Difficulties in Emotion Regulation (DERS-E), Arousal scores and LPP amplitudes from the experimental conditions. The figure presents a correlation matrix visualizing the relationships between variables. Each cell in the matrix provides a Spearman Rho correlation coefficient, with the scale and intensity of the color indicating the strength and direction of the correlation. Darker shades of blue indicate lower positive or negative correlations, while darker shades of yellow denote stronger positive correlations. Asterisks denote levels of statistical significance, with *p* < 0.05*, *p* < 0.001** FDR corrected.

The correlation analyses between attachment dimensions (ECR-12), emotional dysregulation (DERS-E), reports of arousal, and LPP (μV) amplitudes during the four experimental conditions (Figure 4 and Supplementary Table S12) revealed a significant positive relationship between the level of attachment avoidance and Emotional Inattention, Emotional Confusion, and DERS-E Total as measured by the DERS-E scale. Similarly, the attachment anxiety dimension was positively and significantly associated with the dimensions of Emotional Rejection, Lack of Emotional Control, Emotional Interference, and DERS-E Total as assessed by the DERS-E scale. No correlation was found between Emotional Rejection, Lack of Emotional Control, and Emotional Interference with the attachment avoidance dimension, nor between Emotional Inattention, Emotional Confusion and the attachment anxiety dimension.

In the case of arousal levels across the four experimental conditions, a positive and significant relationship was identified between the arousal score in Natural Negative, Reappraise, and Suppress conditions with the Emotional Interference dimension from the DERS-E scale.

Lastly, no significant correlations were discovered between the LPP, and the levels of arousal reported during the four experimental conditions. There was also no significant correlation with the attachment dimensions (ECR-12) nor with the dimensions of the DERS-E.

Overall, the correlation matrix provides a comprehensive overview of the interplay between neural responses to emotional stimuli, as measured by the LPP, and psychological constructs such as attachment anxiety, avoidance, and facets of emotional dysregulation. The patterns of correlation elucidate the complex relationships and potential pathways through which attachment orientations may influence or be influenced by the ability to regulate emotions.

The moderation analyses revealed a significant main effect of the level of attachment anxiety on Emotional Rejection, Lack of Emotional Control, Emotional Interference, and Emotional Confusion as measured by the DERS-E scale. No main effect of attachment anxiety was found on emotional Inattention. As expected, a significant moderating effect of the LPP ROI-Reapp amplitude was discovered in the relationship between the level of attachment anxiety and Lack of Emotional Control (Table 3).

**Table 3:** Moderating effect of LPP during reappraisal on the association between attachment anxiety and difficulties in emotion regulation.

	Emotional rejection DERS-E				
	*β*	SEM	CI 95%	*Z*	*p*
ECR-12-ANX	**2.83**	**0.75**	**1.36–4.30**	**3.78**	< 0.001**
LPP Reapp	−0.19	0.37	−0.92 – 0.53	−0.52	0.601
ECR12-A × LPP Reapp	−0.07	0.29	−0.65 – 0.50	−0.26	0.792

	Lack of Emotional Control DERS-E				
	*β*	SEM	CI 95%	*Z*	*p*
ECR-12-ANX	**2.36**	**0.52**	**1.34–3.38**	**4.54**	< 0.001**
LPP Reapp	−0.07	0.25	−0.58 – 0.42	−0.30	0.762
ECR-12-ANX × LPP Reapp	**0.47**	**0.20**	**0.06–0.87**	**2.28**	0.022*

	Emotional Interference DERS-E				
	*β*	SEM	CI 95%	*Z*	*p*
ECR-12-ANX	**2.31**	**0.41**	**1.50–3.13**	**5.57**	< 0.001**
LPP Reapp	0.10	0.20	−0.30 – 0.50	0.50	0.616
ECR-12-ANX × LPP Reapp	0.01	0.16	−0.31 – 0.33	0.06	0.950

	Emotional Innatention DERS-E				
	*β*	SEM	CI 95%	*Z*	*p*
ECR-12-ANX	−0.15	0.44	−1.02 – 0.70	−0.35	0.723
LPP Reapp	0.07	0.21	−0.35 – 0.50	0.34	0.733
ECR-12-ANX x LPP Reapp	0.12	0.17	−0.21 – 0.46	0.71	0.475

	Emotional Confusion DERS-E				
	*β*	SEM	CI 95%	*Z*	*p*
**ECR-12-ANX**	**0.69**	**0.25**	**0.18–1.20**	**2.66**	0.008*
LPP Reapp	0.07	0.12	−0.17 – 0.33	0.61	0.542
ECR-12-ANX x LPP Reapp	0.09	0.10	−0.10 – 0.29	0.91	0.358

β (Beta), estimated coefficient for each predictor variable in the model; SEM, standard error of mean; CI, confidence interval; Z, *Z*-value or *Z*-score, which represents the number of standard deviations the coefficient estimate is from zero; *p* < 0.05*; *p* < 0.001**. Bold values indicate statistically significant results.

**Figure 5:**
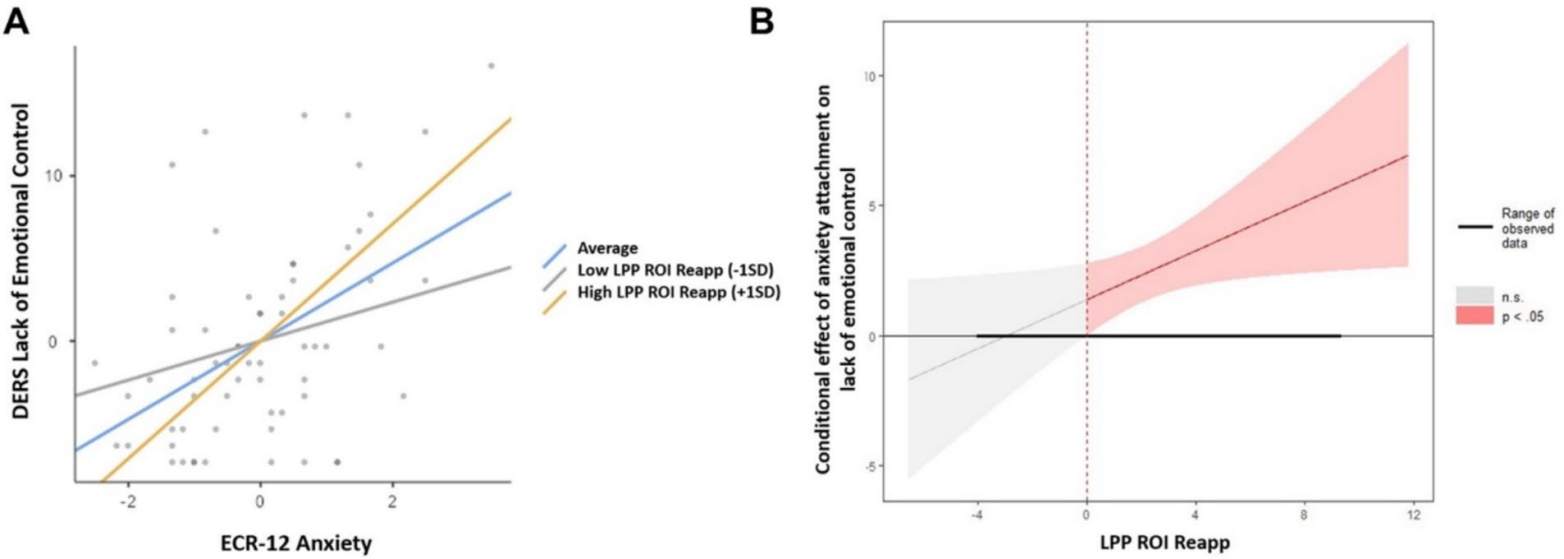
Association between attachment anxiety and lack of emotional control categorized by the LPP amplitude during Reappraise condition and Johnson-Neyman Diagram. (A) Illustrates the relationship between attachment anxiety (ECR-12 Anxiety) and Lack of Emotional Control (DERS-E). The *x*-axis represents standardized scores of attachment anxiety, while the *y*-axis quantifies the difficulties in emotional control. Three distinct lines represent the mean association (Average) and the associations at one standard deviation above (High LPP ROI Reapp [+1SD]) and below (Low LPP ROI Reapp [-1SD]) the mean LPP amplitude measured during the Reappraise condition of the emotion regulation task. These lines were calculated from a moderation analysis, where the LPP amplitude during Reappraise acts as the moderator variable. The blue line (Average) shows the mean relationship between attachment anxiety and lack of emotional control. The yellow line (High LPP ROI Reapp [+1SD]) indicates the relationship when the LPP amplitude during Reappraise is higher than average, showing a steeper slope, which suggests that individuals with higher LPP amplitudes have a stronger positive relationship between attachment anxiety and lack of emotional control. The gray line (Low LPP ROI Reapp [–1SD]) represents the relationship when the LPP amplitude is lower than average, demonstrating a less steep slope, which implies a weaker association between attachment anxiety and lack of emotional control for individuals with lower LPP amplitudes. The individual data points reflect participants’ scores on both variables, providing a visual representation of the distribution and variability within the sample. The figure suggests that the amplitude of the LPP during the Reappraise condition may moderate the strength of the association between attachment anxiety and emotional control difficulties. (B) Illustrates the moderating effect of LPP during reappraisal on the association between attachment anxiety level and Lack of Emotional Control. The x-axis indicates the amplitude of LPP measured during the reappraisal (LPP ROI Reapp). The *y*-axis denotes the conditional effect of attachment anxiety on lack of emotional control. The solid black line represents the conditional effect at different values of LPP ROI Reap. This effect becomes statistically significant (*p* < 0.05) when the LPP ROI Reapp surpasses the zero point, as indicated by the vertical dotted line. The shaded red area shows the range of observed data and highlights the values of LPP ROI Reapp where the effect of attachment anxiety on lack of emotional control is significant. The shaded gray area represents non-significant (n.s.) values, where the conditional effect of attachment anxiety on lack of emotional control is not statistically significant. The figure elucidates that as the amplitude of the LPP during reappraisal increases, the impact of attachment anxiety on emotional control difficulties becomes statistically significant, indicating a positive moderation by the LPP. Conversely, at lower amplitudes of the LPP (to the left of the zero point), the relationship does not reach statistical significance. This visualization aids in understanding the point at which LPP begins to significantly influence the association between attachment anxiety and emotional control difficulties.

The analysis of simple effects (Figure 5A) displays an enhancing moderating effect on the studied relationship. Specifically, when the amplitude of the LPP ROI-Reapp is high, the relationship between attachment anxiety and Lack of Emotional Control becomes stronger. Conversely, when the amplitude of the LPP ROI-Reapp is low, this relationship is not significant.

Regarding the attachment avoidance dimension, a direct effect was observed on Emotional Rejection, Emotional Inattention, and Emotional Confusion. There was no significant effect on the Lack of Emotional Control and Emotional Interference DERS-E dimensions, nor was there a moderating effect of the LPP ROI-Reapp on the relationship between attachment avoidance and the difficulties in emotion regulation dimensions of the DERS-E (Table 4).

**Table 4:** Moderating effect of LPP during reappraisal on the association between attachment avoidance and difficulties in emotion regulation.

	Emotional rejection DERS-E				
	*β*	SEM	CI 95%	*Z*	*p*
ECR-12-AVD	**3.00**	**0.90**	**1.22–4.78**	**3.31**	< 0.001**
LPP Reapp	−0.18	0.37	−0.92 – 0.55	−0.48	0.630
ECR-12-AVD × LPP Reapp	−0.15	0.33	−0.81 – 0.49	−0.47	0.638

	Lack of emotional control DERS-E				
	*β*	SEM	CI 95%	*Z*	*p*
ECR-12-AVD	0.88	0.72	−0.54 – 2.31	1.21	0.226
LPP ROI-Reap	0.01	0.92	−1.79 – 1.83	0.01	0.985
ECR12-AVD × LPP Reapp	1.74	1.04	−0.31 – 3.80	1.66	0.096

	Emotional interference DERS-E				
	*β*	SEM	CI 95%	*Z*	*p*
ECR-12-AVD	1.00	0.59	−0.15 – 2.16	1.70	0.089
LPP ROI-Reapp	0.14	0.24	−0.33 – 0.63	0.60	0.543
ECR12-AVD × LPP Reapp	−0.00	0.21	−0.42 – 0.42	−0.01	0.987

	Emotional inattention DERS-E				
	*β*	SEM	CI 95%	*Z*	*p*
ECR-12-AVD	**1.80**	**0.41**	**0.87–2.72**	**3.82**	< 0.001**
LPP ROI-Reapp	−0.12	0.19	−0.50 – 0.26	−0.62	0.535
ECR-12-AVD × LPP Reapp	0.32	0.17	−0.01 – 0.66	1.87	0.061

	Emotional confusion DERS-E				
	*β*	SEM	CI 95%	*Z*	*p*
ECR-12-AVD	**1.19**	**0.29**	**0.62–1.76**	**4.11**	< 0.001**
LPP ROI-Reapp	0.03	0.12	−0.20 – 0.27	0.28	0.775
ECR-12-AVD × LPP Reapp	−0.00	0.10	−0.21 – 0.20	−0.02	0.978

β (Beta), estimated coefficient for each predictor variable in the model; SEM, standard error of mean; CI, confidence interval; Z, *Z*-value or *Z*-score, which represents the number of standard deviations the coefficient estimate is from zero; *p* < 0.05*; *p* < 0.001**. Bold values indicate statistically significant results.

The Johnson-Neyman diagram (Figure 5B) illustrates that the association between attachment anxiety and the Lack of Emotional Control intensifies as the amplitude of the LPP during the reappraisal condition (LPP ROI Reapp) increases. Conversely, if the LPP amplitude decreases below the zero level, the relationship between attachment anxiety and Lack of Emotional Control is not significant.

## 4 Discussion

This study delved into the relationship between attachment orientations and emotion regulation, focusing on the moderating role of the LPP in the dynamic between attachment insecurity and emotional dysregulation, specifically lack of emotional control. Our findings elucidate the interplay between neurophysiological markers and psychological constructs, contributing to the broader understanding of emotional processing in the context of attachment theory.

The data indicate that individuals with elevated attachment anxiety exhibit heightened difficulties in emotion regulation when the LPP amplitude during a reappraisal task is high. This suggests that the LPP, a neural correlate of emotional processing (Hajcak et al., 2010), may serve as a magnifying lens, amplifying the emotional regulation challenges faced by those with higher attachment anxiety. It is noteworthy that this pattern of difficulty is particularly pronounced when individuals are engaged in the cognitive effort of reappraisal, implying that the very strategies intended to mitigate emotional distress may, under certain neural conditions, exacerbate it (Thiruchselvam et al., 2011). Our results also suggest that larger LPP amplitudes in individuals with higher attachment anxiety may reflect reappraisal failure rather than low reappraisal efficiency. While low reappraisal efficiency implies that the strategy is applied but not effectively reducing emotional distress, reappraisal failure indicates a more complete inability to implement the strategy, resulting in heightened emotional responses. Further studies may clarify this distinction, which could highlight the severity of emotion regulation difficulties in those with higher attachment anxiety.

In contrast, when the LPP amplitude is low, these challenges appear to diminish or not be present, highlighting the potential for certain neural profiles to confer resilience against the emotional regulation difficulties typically associated with attachment anxiety. This finding is intriguing, as it suggests the existence of a neural threshold below which the typical emotional challenges associated with attachment anxiety are not activated or are less impactful (Mikulincer and Shaver, 2016).

Importantly, our results did not demonstrate a similar moderating effect of the LPP on the relationship between attachment avoidance and emotional dysregulation. This could imply that the emotional regulation difficulties associated with attachment avoidance are less susceptible to modulation by the immediate neural responses during emotion regulation tasks or that these relationships may be influenced by other neural mechanisms not captured by the LPP. Additionally, this finding may be indicative of the unique characteristics of attachment avoidance, which is often associated with the deactivation of the attachment system, particularly in contexts involving interpersonal closeness and intimacy. Individuals with higher avoidance attachment may have developed a neural processing bias that minimizes behavioral responses to avoid the discomfort associated with proximity (Fraley and Shaver, 2000; Gillath et al., 2005). Therefore, the LPP amplitudes in these individuals might not exhibit significant fluctuations during emotion regulation tasks that do not involve interpersonal content.

Moreover, our study reaffirms the complexity of the attachment system and its manifestation in adulthood. The strong associations between attachment anxiety and dimensions such as emotional rejection, lack of emotional control, and emotional confusion underscore the pervasive impact of early attachment experiences on adult emotional life. Our research further underscores the value of integrating neurophysiological measures, like the LPP, to deepen our understanding of these psychological phenomena.

The implications of this research are manifold. Clinically, these findings can inform therapeutic approaches by highlighting the potential utility of neurofeedback or other interventions aimed at modulating neural responses to enhance emotion regulation strategies in individuals with attachment anxiety (Barreiros et al., 2019; Dehghani et al., 2020; Herwig et al., 2019; Linhartová et al., 2019; Yu et al., 2021; Zaehringer et al., 2019). From a theoretical standpoint, the results support a more nuanced model of attachment that accounts for individual differences in neurophysiological reactivity and their contributions to emotional regulation capacities (Gross, 2015).

One limitation of our study is the sample size, comprising 63 Chilean Latin-American adults. A larger sample could provide a more robust and generalizable understanding of the interactions between attachment orientations, emotion regulation difficulties, and neural responses. Future research with larger and more diverse samples is essential to validate and expand upon our findings. Another limitation is the variability in how participants interpret and apply suppression and reappraisal strategies during the emotion regulation task. Despite clear instructions and pre-task training, individual differences in understanding and implementing these strategies can introduce noise into the data, making it challenging to draw definitive conclusions about their effectiveness (Domic-Siede et al., 2024a). For instance, different approaches to cognitive reappraisal among participants can lead to variations in emotional outcomes not solely due to attachment orientations. Furthermore, our design approached suppression and reappraisal as mutually exclusive strategies, which is not reflective of real-world scenarios where multiple strategies are often employed concurrently (Gross, 2024). This structured approach may not capture the nuanced ways these strategies naturally occur and interact. The variability in strategy use adds complexity, as enforcing and verifying the exclusive use of one strategy in a controlled environment is challenging (Koval and Kalokerinos, 2024; Bargh and Chartrand, 2000). Future research could benefit from methodologies like real-time reporting or ecological momentary assessment to better understand how individuals navigate and combine different emotion regulation strategies.

Future research should continue to explore the role of other ERPs and neural mechanisms in emotion regulation and attachment, as well as investigate how these processes unfold across different emotional contexts and individual histories. Longitudinal studies could provide additional insights into how these neural patterns develop over time and their potential as predictive markers for emotional regulation capabilities.

In conclusion, our study represents a significant step forward in the neuroscientific exploration of attachment and emotion regulation. By integrating psychological theory with neurophysiological data, we have uncovered new dimensions of the human emotional experience and paved the way for future investigations into the biological underpinnings of our social bonds and emotional lives.

## Data Availability

The raw data supporting the conclusions of this article will be made available by the authors, without undue reservation.

## Footnotes

^1^http://www.gpower.hhu.de/

^2^www.neurobs.com

^3^https://www.antneuro.com/
