# Supplementary material for: The moderating role of the late positive potential in the link between attachment anxiety and emotion regulation difficulties
Source: Front Psychol. 2024 Nov 13;15:1360366. doi: 10.3389/fpsyg.2024.1360366 (PMC11598532; doi:10.3389/fpsyg.2024.1360366)
Supplement: Supplementary file 1 [file Table_1.DOCX]

**Supplementary Material**

**Table S1. Mental Health Disorders in the Total Sample**

| Reported Psychiatric Diagnosis | n |
| --- | --- |
| Anxiety | 7 |
| Depression | 1 |
| Anxiety and Depression | 7 |
| Total | 15 |

**Table S2. Examples of items from DERS-E’ Dimensions** (Guzmán-González et al., 2014)

| **Item** |
| --- |
| **Dimension 1: Emotional Rejection** |
| 10. When I feel bad, I get angry with myself for feeling that way. |
| 11. When I feel bad, I am ashamed of feeling that way. |
| 20. When I feel bad, I feel guilty for feeling that way. |
| **Dimension 2:** **Lack of Emotional Control** |
| 3. I experience my emotions as something overwhelming and out of control. |
| 13. When I feel bad, I lose control. |
| 22. When I feel bad, I have trouble controlling my behavior. |
| **Dimension 3: Emotional Interference** |
| 12. When I feel bad, I have difficulty completing tasks. |
| 16. When I feel bad, I find it difficult to concentrate on other things. |
| 24. When I feel bad, I have trouble thinking about anything else. |
| **Dimension 4: Emotional Inattention** |
| 1. I perceive my feelings clearly*. |
| 2. I pay attention to how I feel*. |
| 6. I am aware of my feelings*. |
| **Dimension 5: Emotional Confusion** |
| 4. I have no idea how I feel. |
| 5. I have trouble understanding my feelings. |
| 8. I am confused about what I feel. |

* = inverse items

**Dimension 1. Emotional Rejection:** This factor measures how individuals react to their negative emotions with self-rejection. It includes feelings of anger, shame, and guilt towards oneself when experiencing negative emotions. It reflects a tendency to internalize and negatively evaluate one's own emotional experiences.

**Dimension 2. Lack of Emotional Control:** This factor assesses the extent to which individuals feel out of control when experiencing negative emotions. It measures the difficulty in maintaining control over emotions and behaviors, indicating a high level of emotional dysregulation and the overwhelming nature of these emotions.

**Dimension 3. Emotional Interference:** This factor measures the degree to which negative emotions interfere with cognitive and behavioral functioning. It includes difficulties in concentrating, completing tasks, and thinking about anything other than the negative emotions being experienced.

**Dimension 4. Emotional Inattention:** This factor evaluates the awareness and clarity individuals have about their own emotions. It measures the extent to which individuals are attentive to, and understand, their emotional states. Lower scores in this factor suggest a lack of emotional awareness and clarity.

**Dimension 5: Emotional Confusion:** This factor assesses the confusion and lack of understanding individuals have regarding their emotions. It reflects difficulty in identifying and making sense of emotional experiences, often leading to an overall sense of confusion about one's emotional state.

**Table S3. Selected IAPS pictures per condition (Lang et al., 2005)**

|  | Experimental Conditions | | | |
| --- | --- | --- | --- | --- |
| N° | Natural-neg | Suppress | Reappraise | Natural-neu |
| 1 | 2683 | 2095 | 2130 | 7009 |
| 2 | 2703 | 2205 | 2700 | 7011 |
| 3 | 2710 | 2900 | 2751 | 7012 |
| 4 | 2717 | 6211 | 2811 | 7018 |
| 5 | 3220 | 6231 | 3550 | 7042 |
| 6 | 3230 | 6250 | 6260 | 7045 |
| 7 | 3500 | 6312 | 6350 | 7061 |
| 8 | 6300 | 6560 | 6530 | 2190 |
| 9 | 6315 | 6563 | 6561 | 2215 |
| 10 | 6360 | 6838 | 6562 | 2359 |
| 11 | 6540 | 9041 | 6825 | 2499 |
| 12 | 6550 | 9250 | 6834 | 2518 |
| 13 | 6840 | 9421 | 9050 | 2521 |
| 14 | 9429 | 9427 | 9419 | 2593 |
| 15 | 9900 | 9435 | 9425 | 2594 |

Natural-neg: Natural condition containing negative valence pictures; Natural-neu: Natural condition containing neutral valence pictures.

**Table S4. Descriptive statistics of the selected IAPS pictures per condition.**

|  | Arousal | |
| --- | --- | --- |
|  | Mean | SD |
| Natural-neg | 6.09 | 0.59 |
| Suppress | 5.76 | 0.81 |
| Reappraise | 5.83 | 0.81 |
| Natural-neu | 3.48 | 0.48 |

Natural-neg: Natural condition containing negative valence pictures; Natural-neu: Natural condition containing neutral valence pictures. SD = Standard Deviation.

**Table S5.** **Multiple comparison test for the arousal of IAPS pictures between conditions**

| Tukey’s test | Mean Diff. | 95.00% CI of diff. | Summary | Adjusted p-value |
| --- | --- | --- | --- | --- |
| Natural-Neg vs. Suppress | 0.3240 | -0.3415 to 0.9895 | ns | 0.5736 |
| Natural-Neg vs. Reappraise | 0.2633 | -0.4022 to 0.9288 | ns | 0.7222 |
| **Natural-Neg vs. Natural-Neu** | **2.607** | **1.942 to 3.273** | ******** | **<0.0001** |
| Suppress vs. Reappraise | -0.06067 | -0.7262 to 0.6048 | ns | 0.9950 |
| **Suppress vs. Natural-Neu** | **2.283** | **1.618 to 2.949** | ******** | **<0.0001** |
| **Reappraise vs. Natural-Neu** | **2.344** | **1.679 to 3.009** | ******** | **<0.0001** |

Natural-neg: Natural condition containing negative valence pictures; Natural-neu: Natural condition containing neutral valence pictures.; ns: not significant.

**Table S6. Correlations between ECR-12 Dimensions and STAI Dimensions**

| **Correlations** | **Spearman rho** | **95% CI** | **P value** |
| --- | --- | --- | --- |
| ECR-12 ANX vs. STAI-S | 0.11 | -0.15 to 0.36 | 0.3811 |
| ECR-12 ANX vs. STAI-T | 0.23 | -0.03 to 0.46 | 0.0708 |
| ECR-12 AVD vs. STAI-S | 0.11 | -0.15 to 0.36 | 0.3733 |
| ECR-12 AVD vs. STAI-T | 0.17 | -0.09 to 0.40 | 0.1961 |

This table summarizes the Spearman correlation analysis between the ECR-12 Anxiety (ANX) and Avoidance (AVD) Attachment scales and the STAI-T and STAI-S scores.

**Table S7.** **Descriptive Statistics of the Final Number of Trials per Condition**

| **Condition** | **Number of values** | **Minimum** | **25% Percentile** | **Median** | **75% Percentile** | **Maximum** | **Mean** | **Std. Deviation** | **Std. Error of Mean** | **Lower 95% CI** | **Upper 95% CI** | **Mean ranks** |
| --- | --- | --- | --- | --- | --- | --- | --- | --- | --- | --- | --- | --- |
| Negative | 63 | 9.0 | 14.0 | 15.0 | 15.0 | 15.0 | 14.44 | 1.0120 | 0.12760 | 14.19 | 14.70 | 2.421 |
| Neutral | 63 | 8.0 | 14.0 | 15.0 | 15.0 | 15.0 | 14.52 | 1.0600 | 0.13360 | 14.26 | 14.79 | 2.548 |
| Reappraise | 63 | 9.0 | 14.0 | 15.0 | 15.0 | 15.0 | 14.41 | 1.1590 | 0.14600 | 14.12 | 14.70 | 2.397 |
| Suppress | 63 | 12.0 | 14.0 | 15.0 | 15.0 | 15.0 | 14.63 | 0.7252 | 0.09136 | 14.45 | 14.82 | 2.635 |

**Table S8. Friedman Test for the Final Number of Trials per Condition**

| **Test** | **P value** | **Number of groups** | **Friedman statistic** |
| --- | --- | --- | --- |
| Friedman test | 0.3089 | 4 | 3.593 |

**Table S9. Dunn's Multiple Comparisons Test for the Final Number of Trials per Condition**

| **Comparison** | **Rank sum diff.** | **Significant?** | **Summary** | **Adjusted P Value** |
| --- | --- | --- | --- | --- |
| Negative vs. Neutral | -8.0 | No | ns | >0.9999 |
| Negative vs. Reappraise | 1.5 | No | ns | >0.9999 |
| Negative vs. Suppress | -13.5 | No | ns | >0.9999 |
| Neutral vs. Reappraise | 9.5 | No | ns | >0.9999 |
| Neutral vs. Suppress | -5.5 | No | ns | >0.9999 |
| Reappraise vs. Suppress | -15.0 | No | ns | >0.9999 |

**Table S10.** Moderating Effect of LPP During Suppress on the Association Between Attachment Anxiety and Difficulties in Emotion Regulation.

|  | **Emotional Rejection DERS-E** | | | | |
| --- | --- | --- | --- | --- | --- |
|  | **β** | **SEM** | **IC 95%** | **Z** | **p** |
| ECR-12-ANX | 2.82 | 0.74 | 1.36 – 4.29 | 3.77 | <.001** |
| LPP Supp | 0.09 | 0.38 | -0.65 – 0.84 | 0.24 | 0.806 |
| ECR12-A x LPP Supp | 0.24 | 0.29 | -0.33 – 0.83 | 0.82 | 0.408 |
|  | **Lack of Emotional Control DERS-E** | | | | |
|  | **Β** | **SEM** | **IC 95%** | **Z** | **p** |
| ECR-12-ANX | 2.39 | 0.52 | **1.36 – 3.42** | 4.54 | < .001 |
| LPP Supp | -0.36 | 0.26 | -0.88 – 0.16 | -1.35 | 0.177 |
| ECR-12-ANX x LPP Supp | 0.24 | 0.21 | -0.16 – 0.66 | 1.18 | 0.240 |
|  | **Emotional Interference DERS-E** | | | | |
|  | **β** | **SEM** | **IC 95%** | **Z** | **p** |
| ECR-12-ANX | **2.34** | **0.42** | **1.52 – 3.16** | **5.63** | **< .001**** |
| LPP Supp | 0.05 | 0.21 | -0.46 – 0.36 | -0.24 | 0.812 |
| ECR-12-ANX x LPP Supp | 0.03 | 0.17 | -0.28 – 0.36 | 0.23 | 0.816 |
|  | **Emotional Innatention DERS-E** | | | | |
|  | **β** | **SEM** | **IC 95%** | **Z** | **p** |
| ECR-12-ANX | -0.10 | 0.43 | 0.93 – 0.73 | -0.24 | 0.810 |
| LPP Supp | 0.11 | 0.22 | -0.31 – 0.54 | 0.51 | 0.607 |
| ECR-12-ANX x LPP Supp | 0.40 | 0.17 | 0.07 – 0.07 | 2.40 | 0.016* |
|  | **Emotional Confusion DERS-E** | | | | |
|  | **β** | **SEM** | **C.I. 95%** | **Z** | **p** |
| ECR-12-ANX | 0.71 | 0.26 | 0.21 – 1.22 | 2.80 | 0.005* |
| LPP Supp | 0.13 | 0.13 | -0.13 – 0.38 | 0.98 | 0.328 |
| ECR-12-ANX x LPP Supp | 0.17 | 0.10 | -0.03 – 0.36 | 1.63 | 0.104 |

β (Beta) = estimated coefficient for each predictor variable in the model; SEM = Standard Error of Mean; CI = Confidence Interval; Z = Z-value or Z-score, which represents the number of standard deviations the coefficient estimate is from zero; p<.05*, p<.001**

**Table S11.** Moderating Effect of LPP During Suppress on the Association Between Attachment Avoidance and Difficulties in Emotion Regulation.

|  | **Emotional Rejection DERS-E** | | | | |
| --- | --- | --- | --- | --- | --- |
|  | **β** | **SEM** | **IC 95%** | **Z** | **p** |
| ECR-12-AVD | 3.02 | 0.90 | 1.25 – 4.80 | 3.34 | < .001** |
| LPP Supp | 0.00 | 0.39 | -0.76 – 0.77 | 0.01 | 0.990 |
| ECR-12-AVD x LPP Supp | -0.15 | 0.36 | -0.86 – 0.56 | -0.41 | 0.679 |
|  | **Lack of Emotional Control DERS-E** | | | | |
|  | **β** | **SEM** | **IC 95%** | **Z** | **p** |
| ECR-12-AVD | 0.70 | 0.70 | -0.68 – 2.08 | 1.00 | 0.320 |
| LPP ROI- Supp | -0.38 | 0.30 | -0.98 - 0.21 | -1.26 | 0.206 |
| ECR12-AVD x LPP Supp | 0.30 | 0.28 | -0.25 – 0.85 | 1.06 | 0.287 |
|  | **Emotional Interference DERS-E** | | | | |
|  | **β** | **SEM** | **IC 95%** | **Z** | **p** |
| ECR-12-AVD | 1.07 | 0.59 | -0.08 – 2.22 | 1.82 | 0.068 |
| LPP ROI- Supp | -0.04 | 0.25 | -0.54 – 0.44 | -0.19 | 0.849 |
| ECR12-AVD x LPP Supp | -0.11 | 0.23 | -0.57 – 0.35 | -0.47 | 0.636 |
|  | **Emotional Inattention DERS-E** | | | | |
|  | **β** | **SEM** | **IC 95%** | **Z** | **p** |
| ECR-12-AVD | 1.60 | 0.48 | 0.69 – 2.52 | 3.43 | < .001** |
| LPP ROI- Supp | -0.01 | 0.20 | -0.41 – 0.37 | -0.08 | 0.935 |
| ECR-12-AVD x LPP Supp | 0.35 | 0.19 | -0.01 – 0.71 | 1.86 | 0.062 |
|  | **Emotional Confusion DERS-E** | | | | |
|  | **β** | **SEM** | **IC 95%** | **Z** | **p** |
| ECR-12-AVD | 1.18 | 0.29 | 0.62 – 1.74 | 4.14 | < .001** |
| LPP ROI- Supp | 0.08 | 0.12 | -0.16 – 0.32 | 0.65 | 0.516 |
| ECR-12-AVD x LPP Supp | 0.14 | 0.11 | -0.08 – 0.36 | 1.21 | 0.224 |

β (Beta) = estimated coefficient for each predictor variable in the model; SEM = Standard Error of Mean; CI = Confidence Interval; Z = Z-value or Z-score, which represents the number of standard deviations the coefficient estimate is from zero; p<.05*, p<.001**

**Table S12. FDR corrected p-values of the correlation matrix**

|  | **1** | **2** | **3** | **4** | **5** | **6** | **7** | **8** | **9** | **10** | **11** | **12** | **13** | **14** | **15** | **16** |
| --- | --- | --- | --- | --- | --- | --- | --- | --- | --- | --- | --- | --- | --- | --- | --- | --- |
| **1. ECR-12-AVD** | - | 0.1231 | 0.3024 | 0.3044 | 0.1457 | 0.0129* | 0.0169* | 0.0046** | 0.5400 | 0.5291 | 0.9461 | 0.7587 | 0.6400 | 0.5400 | 0.3024 | 0.9461 |
| **2. ECR-12-ANX** | 0.1231 | - | 0.0046** | 0.0046** | 0.0046** | 0.9680 | 0.0353* | 0.0046** | 0.7252 | 0.2320 | 0.3044 | 0.4210 | 0.8267 | 0.4297 | 0.6744 | 0.7226 |
| **3. DERS-ER** | 0.3024 | 0.0046** | - | 0.0046** | 0.0046** | 0.0155* | 0.0046** | 0.0046** | 0.9680 | 0.1320 | 0.2732 | 0.1405 | 0.9461 | 0.8635 | 0.9461 | 0.9680 |
| **4. DERS-LEC** | 0.3044 | 0.0046** | 0.0046** | - | 0.0089** | 0.3434 | 0.0514 | 0.0046** | 0.3120 | 0.3035 | 0.2182 | 0.3434 | 0.9680 | 0.6919 | 0.8267 | 0.3946 |
| **5. DERS-INT** | 0.1457 | 0.0046** | 0.0046** | 0.0089** | - | 0.9461 | 0.2452 | 0.0046** | 0.5950 | 0.0155 | 0.0155* | 0.0327* | 0.8635 | 0.9461 | 0.8267 | 0.9653 |
| **6. DERS-INATT** | 0.0129* | 0.9680 | 0.0155* | 0.3434 | 0.9461 | - | 0.0046** | 0.0046** | 0.8267 | 0.6341 | 0.7226 | 0.6164 | 0.7226 | 0.9680 | 0.5617 | 0.9461 |
| **7. DERS-EC** | 0.0169* | 0.0353* | 0.0046** | 0.0514 | 0.2452 | 0.0046** | - | 0.0046** | 0.2182 | 0.5063 | 0.5657 | 0.5291 | 0.2900 | 0.7070 | 0.4210 | 0.7226 |
| **8. DERS-T** | 0.0046** | 0.0046** | 0.0046** | 0.0046** | 0.0046** | 0.0046** | 0.0046** | - | 0.6810 | 0.0700 | 0.1105 | 0.0973 | 0.7226 | 0.8267 | 0.7214 | 0.9680 |
| **9. Arousal-Nneu** | 0.5400 | 0.7252 | 0.9680 | 0.3120 | 0.5950 | 0.8267 | 0.2182 | 0.6810 | - | 0.0046** | 0.0046** | 0.0046** | 0.9680 | 0.6995 | 0.9461 | 0.9461 |
| **10. Arousal-Nneg** | 0.5291 | 0.2320 | 0.1320 | 0.3035 | 0.0155* | 0.6341 | 0.5063 | 0.0700 | 0.0046** | - | 0.0046** | 0.0046** | 0.7214 | 0.6744 | 0.9461 | 0.8635 |
| **11. Arousal-Reapp** | 0.9461 | 0.3044 | 0.2732 | 0.2182 | 0.0155* | 0.7226 | 0.5657 | 0.1105 | 0.0046** | 0.0046** | - | 0.0046** | 0.4724 | 0.3044 | 0.6744 | 0.6744 |
| **12. Arousal-Supp** | 0.7587 | 0.4210 | 0.1405 | 0.3434 | 0.0327* | 0.6164 | 0.5291 | 0.0973 | 0.0046** | 0.0046** | 0.0046** | - | 0.5662 | 0.3434 | 0.8209 | 0.7226 |
| **13. LPP ROI-Nneg** | 0.6400 | 0.8267 | 0.9461 | 0.9680 | 0.8635 | 0.7226 | 0.2900 | 0.7226 | 0.9680 | 0.7214 | 0.4724 | 0.5662 | - | 0.0046** | 0.0046** | 0.0046** |
| **14. LPP ROI-Neu** | 0.5400 | 0.4297 | 0.8635 | 0.6919 | 0.9461 | 0.9680 | 0.7070 | 0.8267 | 0.6995 | 0.6744 | 0.3044 | 0.3434 | 0.0046** | - | 0.0046** | 0.0046** |
| **15. LPP ROI-Reapp** | 0.3024 | 0.6744 | 0.9461 | 0.8267 | 0.8267 | 0.5617 | 0.4210 | 0.7214 | 0.9461 | 0.9461 | 0.6744 | 0.8209 | 0.0046** | 0.0046** | - | 0.0046** |
| **16. LPP ROI-Supp** | 0.9461 | 0.7226 | 0.9680 | 0.3946 | 0.9653 | 0.9461 | 0.7226 | 0.9680 | 0.9461 | 0.8635 | 0.6744 | 0.7226 | 0.0046** | 0.0046** | 0.0046** | - |

p<.05*, p<.001** FDR corrected.
